# Root-Knot Nematodes Exhibit Strain-Specific Clumping Behavior That Is Inherited as a Simple Genetic Trait

**DOI:** 10.1371/journal.pone.0015148

**Published:** 2010-12-09

**Authors:** Congli Wang, Steven Lower, Varghese P. Thomas, Valerie M. Williamson

**Affiliations:** Department of Nematology, University of California Davis, Davis, California, United States of America; National Institute on Aging, United States of America

## Abstract

Root-knot nematodes are obligate parasites of a wide range of plant species and can feed only on the cytoplasm of living plant cells. In the absence of a suitable plant host, infective juveniles of strain VW9 of the Northern root-knot nematode, *Meloidogyne hapla*, when dispersed in Pluronic F-127 gel, aggregate into tight, spherical clumps containing thousands of worms. Aggregation or clumping behavior has been observed in diverse genera in the phylum Nematoda spanning free-living species such as *Caenorhabditis elegans* as well as both plant and animal parasites. Clumping behavior differs between strains of *M. hapla* and occurs with other species within this genus where strain-specific differences in clumping ability are also apparent. Exposure of *M. hapla* juveniles to a gradient formed using low levels of cyanide promotes formation of clumps at a preferred cyanide level. Analysis of F2 lines from a cross of *M. hapla* strains that differ in clump-forming behavior reveals that the behavior segregates as a single, major locus that can be positioned on the genetic map of this nematode. Clumping behavior may be a survival strategy whose importance and function depend on the niche of the nematode strain or species.

## Introduction

Root-knot nematodes (*Meloidogyne* spp.) are obligate endoparasites of a wide range of plant species and globally cause large crop yield losses [1], [2]. The infective stage, which is the second stage juvenile (J2), hatches from an egg in the soil and must find a host and establish a feeding site in order to survive. The J2 typically enters the host root in the zone of elongation then migrates to the vascular cylinder to establish a feeding site and complete its life cycle. Little is known about the behavior and survival of the infective juvenile. Many nematodes, including most plant parasitic species, inhabit the soil for at least part of their life cycle. Natural nematode behavior has been particularly difficult to study in this opaque, non-uniform environment [3]–[5]. As an attempt to gain insights into the behavior of pre-infective J2s, we developed a Petri dish assay using Pluronic F-127 (PF-127), a clear, non-toxic co-polymer that as a 23% aqueous solution is liquid at 15°C and solidifies into a soft gel at room temperature [6]. Nematodes suspended in PF-127 gel, rather than being limited to the surface as with agar plates, can respond to signals perceived in three dimensions. Movement of nematodes to root tips can be monitored as well as changes in behavior as they approach the zone of elongation where they penetrate the root. Because stable chemical gradients form in PF-127, we were able to show that root-knot nematode J2 accumulate at pH between 4.5 and 5.5, consistent with the low pH of the zone of elongation relative to other regions of the root [7].

A particularly interesting finding was that, when no seedling was included, J2 of *M. hapla* strain VW9, *M. javanica* strain VW4, and *M. incognita* strain 557R aggregate into tightly packed spherical clumps by two days after being uniformly suspended in the PF-127 gel [6]. The clumping behavior was accelerated by increasing the nematode concentration or by putting a coverslip on the gel surface, suggesting that low oxygen or a volatile signal from the nematode was involved. Interestingly, we found that clumps dispersed when a root of an appropriate host was added.

The obligate parasitism and long life cycle of plant parasitic nematodes compared to *C. elegans*, have hindered research approaches aimed at understanding the genes responsible for behavior and parasitism. However, recent advances have increased the feasibility of these goals. Genome sequences are now available for the root-knot nematode species *M. incognita* and *M. hapla* [8], [9]. While several of the agriculturally damaging root-knot nematode species, including *M. incognita* and *M. javanica*, reproduce by mitotic parthenogenesis precluding any genetic analysis, many *M. hapla* strains reproduce by a novel mechanism that allows both outcrossing and selfing [2], [10]. This reproduction mechanism has facilitated the production of F2 lines and the generation of a genetic map based on molecular polymorphisms between the parental strains [9], [10]. As a consequence of their novel reproductive mechanism, these F2 lines are homozygous for most markers and thus resemble recombinant inbred lines [10]. Due to the availability of the genome sequence, a genetic map, and phenotypic variability within species, *M. hapla* is emerging as a model plant parasitic nematode. Here we investigate the inheritance in F2 lines of the ability to form clumps and present the first demonstration of the utility of these lines for mapping a biologically significant trait.

## Results

### Clumping behavior differs among root-knot nematode strains and species

We previously found that infective J2 of three different root-knot nematode species (*M. hapla* strain VW9, *M. javanica* strain VW4, and *M. incognita* strain 557R) aggregated into tightly packed spherical clumps by two days after being uniformly suspended in PF-127 [6]. To expand this analysis, 6 inbred *M. hapla* strains that originated from diverse geographic locations and hosts were compared for this behavior (Table 1). Four of these strains (VW9, VW10, VW11, and VW12) formed clumps by the second day. In contrast, the *M. hapla* strain NCS aggregated to some extent but never formed tight clumps, and strain LM did not aggregate at all. Analysis of cultures of closely related *M. javanica* strains VW4 and VW5 that differ in their ability to infect tomato with the resistance gene *Mi-1* found that both formed clumps equally well. However, five *M. incognita* strains initially obtained from different locations and hosts tested differed dramatically in clumping behavior (Table 1). *Meloidogyne incognita* strain VW6 formed multiple clumps in one day, whereas strains Beltran and W1 formed 1–3 clumps, and strain Harmony formed none even after 48 h. As previously noted [6], when a coverslip was placed on the surface of the gel, the clumping was accelerated for each clump-forming strain and was visible under the coverslip within 24 hr (not shown). However, those strains that did not clump were not induced to do so by the addition of a coverslip.

**Table 1:** Comparison of *Meloidogyne* strain clump formation and attraction to cyanide^a^.

Species and strains	Nematode isolate origin:Host and geographic location	Clumps inPF-127 by48 h	2 mM KCN:# J2 within 5 mmat 3 h^b^	2 mM KCN: clump formation by 24 h
*M. hapla*				
VW9	Tomato, CA, USA	Yes	116.8a	Yes
VW10	Hemp, The Netherlands	Yes	65.4b	Yes
VW11	Everlasting flowers, The Netherlands	Yes	74.7b	Yes
VW12	Tomato, CA, USA	Yes	65.7b	Yes
LM	Unknown host, France	No	50.0c	No
NCS	Unknown host, North Carolina, USA	No	48.0c	No
*M. javanica*				
VW4	Unknown, CA, USA	Yes	46.8c	Yes
VW5	Unknown, CA, USA	Yes	50.8c	Yes
*M. incognita*				
VW6	Cotton, CA, USA	Yes	20.3d	No
Beltran	Lima bean, CA, USA	Yes	31.3d	No
Harmony	Grape, CA, USA	No	9.6e	No
557R	Tomato, NC, USA	Yes	30.3d	No
W1	Tomato, CA, USA	Yes	23.6d	No

a Petri dishes contained 300 J2 per ml PF-127 gel.

b Average number of nematodes within 5 mm of the small opening of the dispenser minus the average number of nematodes in the corresponding region with a dispenser containing PF-127 gel alone. Significant differences are indicated by different letters (P<0.05).

### Exposure to cyanide gradients promotes clumping behavior

**Figure 1:**
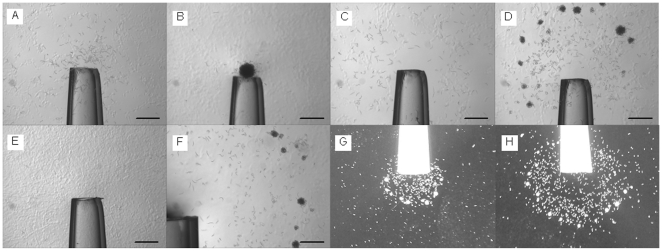
Response of *Meloidogyne hapla* VW9 to potassium cyanide gradients in PF-127 gel. Region of the gel around the small opening of chemical dispenser with 2 mM KCN is shown at 5 h (A) and 24 h (B) after initiation of the assay. Same region when dispenser contains 5 mM KCN is shown at 5 h (C) and 24 h (D). Panel E shows opening of dispenser containing water at 24 hr. Panel F shows a detail of large end of dispenser with 5 mM KCN at 24 hr. Panels G and H are taken at lower magnification with backlighting. In these panels the large end of the dispenser (4 mm diameter) is shown for 2 mM KCN (G) and 5 mM KCN (H) at 24 h after assay initiation. Petri dish containing 600 J2 per ml PF-127 gel was used for each assay. Scale bar in A-F is 1 mm.

In preliminary chemical screens we noted that low concentrations of potassium cyanide (KCN) appeared to attract *M. hapla* VW9 J2 and cause them to form clumps. To investigate this further, chemical dispensers (modified pipet tips; see reference 7) filled with 2 or 5 mM KCN were placed into Petri dishes containing J2 of VW9 dispersed in PF-127 gel. Based on our previous work with pH gradients [7], we anticipated that KCN would form a radial concentration gradient centered on the openings of the dispensers. By 5 h after assay initiation, nematodes could be seen to accumulate at both openings of the chemical dispensers with 2 mM KCN (Figure 1A), and in a region of about 5 mm diameter around the small opening of the dispenser with 5 mM KCN (Figure 1C). By 24 hr, nematodes had aggregated into a compact ball at the small opening of the dispenser (Figure 1B). For the dispenser with 5 mM KCN, the clumps formed a ring centered on the opening (Figure 1D). A circle of clumps was also seen around the large openings of the dispensers with 2 mM or 5 mM KCN with a larger diameter at the latter concentration (Figure 1F-H). For those cases with a ring of clumps, nematodes also accumulated within the circle, but the area closest to the opening of the dispenser was depleted of nematodes (Figure 1D). This novel distribution of J2 was reproducible in multiple replicates with independently hatched nematodes. In controls in which the dispenser contained only PF-127 gel, nematodes remained randomly distributed in the plate (Figure 1E). We measured the cyanide ion concentration in gel samples taken at the inner and outer boundary of the regions with the highest nematode accumulation outside the openings of dispensers with 5 mM KCN using a cyanide-specific electrode at 24 h after assay start. The cyanide concentration at which nematodes accumulated was estimated to be between 15.3±0.93 µM and 22.0±0.60 µM (R^2^ = 90.2). By 48 h, nematode accumulation near the dispenser openings was greatly reduced for all KCN treatments and the patterns were less evident. A likely explanation was that the cyanide concentration was reduced through volatilization.

Since KCN solution has a high pH, we repeated the assay with the nematodes originally suspended in PF-127 gel buffered with 10 mM sodium phosphate, pH 7.0, or 10 mM TRIS MES, pH 7.1. We did not see any difference in response to 2 mM KCN when nematodes were suspended in PF-127 in either of these buffers compare to those suspended in water. In addition, we did not see accumulation or aggregation near openings of dispensers containing only 2 mM sodium phosphate (pH 8.0), 5 mM KCl, or 2 mM KOH, indicating that cyanide ion, and not buffer or potassium ions or elevated pH, was responsible for the attraction.

Because nematodes have been widely reported to be sensitive to cyanide [11]–[13], we examined the response of *M. hapla* VW9 J2 to a series of KCN concentrations in 10 µM increments from 0–110 µM in a 6-well-plate containing 1500 J2s in 5 ml water in each well. Nematodes were observed under microscope after 30 minutes. Nematodes in water displayed typical sinusoidal movement. In 10 and 20 µM KCN movement was slower and nematodes were curled, that is, head and tail were in close proximity resembling the conformation that we have previously observed as nematodes reach the root surface [6]. In 30 µM KCN, most nematodes were straight and did not move. All nematodes were rigid at cyanide concentrations of 40 µM or higher. By 24 h, nematodes had regained motility in all concentrations of cyanide tested, including 110 µM. The same responses were observed in PF-127 gel with this series of KCN concentrations, suggesting that cyanide effects are reversible because cyanide volatilizes.

**Figure 2:**
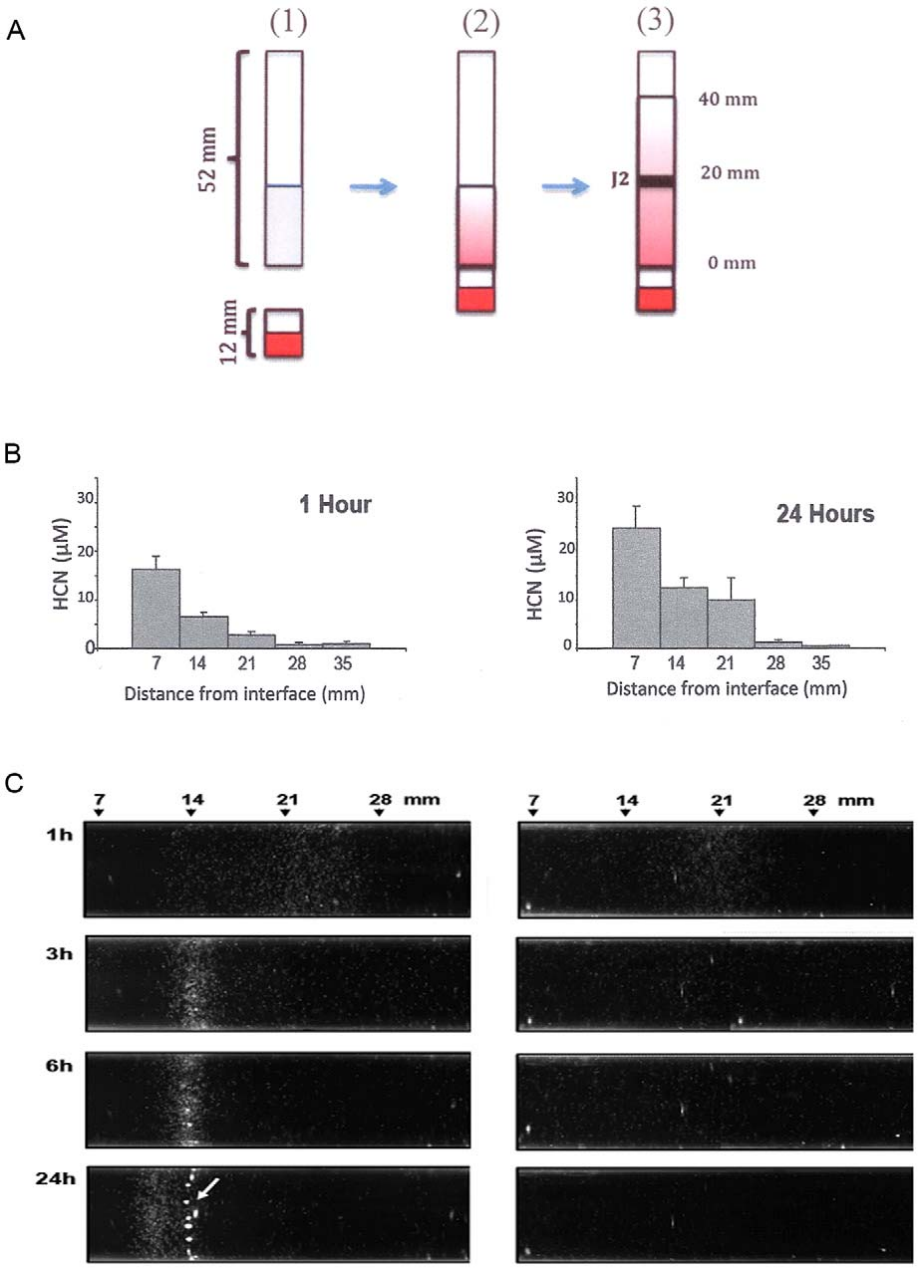
Response of nematodes to cyanide in a closed system. A. Diagram of assay design: (1) 23% PF-127 (grey shading) was added to 52 mm-long polystyrene tubing and KCN (red shading) was added to the donor tube (12-mm long). (2) After gel formed, the two tubes were connected. Hydrogen cyanide was produced in the donor tube then allowed to move across the airspace and to form a gradient upon re-dissolving in PF-127 gel in the assay tube. Gradient was allowed to form for 24 h. (3) J2s in PF-127 were layered into the assay tube and a layer of 23% PF-127 solution was pipetted on top. This addition was considered assay time point 0. B. Cyanide concentration measured at indicated distance from the interface assay tube and airspace of donor tube at 1 and 24 hr after time point 0. C. Tubes were photographed with side lighting to visualize the nematodes by reflected light at indicated times after assay start. A control experiment with no KCN in donor tube is shown on the right. Numbers at top correspond to distance (mm) from the bottom of the assay tube. The left-most 6 mm of the assay tube are not shown due to glare from the side lighting. The white arrow in C points to nematode clumps.

To circumvent volatilization of cyanide, we examined nematode response in a closed system in which the nematodes are exposed to a pre-formed cyanide gradient in a polystyrene tube (Figure 2A). Cyanide concentration was measured at several positions in a parallel assay tube without nematodes at one and 24 h after assay initiation (Figure 2B). By 1 h after assay start, nematodes had moved from the origin, and at 3 h they had formed a band that became tighter by 6 h (Figure 2C). By 24 h, several spherical clumps were visible at 14 mm, which, based on our assays in the control tube, was at ∼12 µM cyanide, similar to the concentration at which clumps formed in the Petri dish assay. A loose aggregation of nematodes continued to move into higher concentrations of cyanide. Many of the nematodes in the leading region at 24 h were observed to be curled in confirmation, rather than sinusoidal. The cyanide concentration in this region was estimated to be between 12 and 25 µM. In control assay tubes without the cyanide gradient, most J2 remained in sinusoidal shape, dispersed throughout the tube, and formed neither bands nor clumps.

### *Meloidogyne* strains differ in response to cyanide

We compared the responses of different root-knot nematode species and strains to KCN gradients in PF-127 gels (Table 1). At 3 hours, VW9 had the greatest accumulation near the dispenser opening of the *M. hapla* strains tested, and strains LM and NCS were significantly less responsive than VW10, 11, and 12. The closely related *M. javanica* strains VW4 and VW5 were significantly less responsive than *M. hapla* strains VW9, 10, 11, or 12. The five *M. incognita* strains tested showed less accumulation than the other species, with *M. incognita* strain Harmony showing the least accumulation. By 24 h, the numbers of nematodes gathered near the dispenser opening were too high to count (>100), and some strains had formed clumps around the opening (Table 1). For *M. hapla* and *M. javanica* strains, the presence or absence of clumps correlated with that seen in plates with PF-127 alone at 48 hr. However, no clumps were seen for any of the five *M. incognita* strains tested even though some of these formed clumps in PF-127 at 48 h.

### Inheritance of clumping trait in *Meloidogyne hapla*

**Figure 3:**
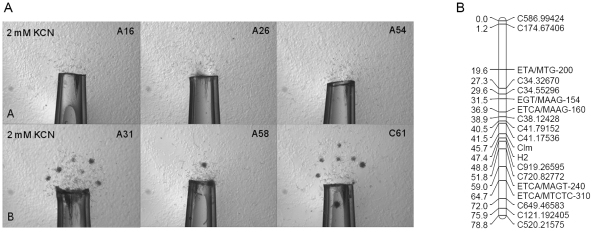
Segregation of clumping behavior in *M. hapla* F2 lines. **A**. Response to dispensers with 2 mM KCN in PF-127 gel 24 h after initiation of assay of F2 lines A16, A26, A54 (non-clumping), and lines A31, A58, and C61 (clumping). Petri dish contained 300 J2 per ml in 20 ml PF-127 gel for each assay. **B**. Map of linkage group 8 with the positions of DNA markers and the Clumping locus (Clm). Size bar in A is 1 mm.

We assessed J2 from greenhouse cultures of 87 F2 lines from a cross of *M. hapla* strains VW8 and VW9 for clump formation in response to cyanide gradients produced around openings of chemical dispensers containing 2 mM KCN in PF-127 gel. Of the 87 lines, 40 were scored as non-clumping, 45 as clumping (Figure 3A). Two lines produced ambiguous results giving small clumps in some replicates, but none in others. In addition, the degree of clumping varied to some extent between replicates in that some consistently produced large clumps whereas other produced only small ones. Nevertheless, using the 85 lines that we were able to score, the Clumping trait (Clm) could be assigned to Linkage Group 8 of the genetic map using JoinMap® 4 (LOD 10.0), and its relative position on the linkage group assigned (Figure 3B).

## Discussion

All assays that attempt to examine natural behavior of microscopic organisms in the laboratory have limitations. However, such assays can lead to discovery of novel, relevant behaviors. PF-127 gel is highly transparent and provides sufficient traction to allow nematodes to move easily resolving difficulties with assays on agar surfaces where nematodes can be trapped in water films. We have found that the formation of tight clumps by root-knot nematodes initially dispersed in PF-127 gel is a remarkably reproducible behavior. We have observed similar clumps on agar plates and in liquid, although in these two media the clumps were noted less consistently. Thus, while clumping is likely a natural phenomenon, the properties of the PF-127 gel, especially its ability to reduce or eliminate convection, may stimulate clumping behavior and stabilize the clumps. We observe three general conformations, sinusoidal, curled (head and tail in close proximity), and straight for nematodes in Pluronic gel. The sinusoidal shape was observed for nematodes undergoing directed movement, whereas the curled shape has been seen previously for nematodes in close proximity to their favored entry sites on host roots [6] and, in the current work, for nematodes in response to cyanide. Nematodes in the curled conformation change position in the gel more slowly than sinusoidal worms. The straight conformation has been observed for nematodes paralyzed by cyanide or other chemicals as well as for dead worms.

Aggregation, clumping and related behaviors have been observed in diverse nematode species, including free-living species and both plant and animal parasites [14]–[18]. For root-knot nematodes, the observations that the rate of clump formation is positively correlated with nematode population density and is accelerated by placement of a coverslip on the gel surface are consistent with the hypotheses that reduced oxygen, increased concentration of a volatile pheromone or contact with a solid surface can initiate or stabilize the clumping [6]. Clumping can also be seen when high numbers of infective J2 reach the zone of elongation of a healthy root, the targeted entry site for this nematode. In this case, signals from the root or from J2 may be signaling the clumping behavior [6].

Perhaps the most studied example of nematode aggregation is as a component of a set of related behaviors including social feeding (aggregation on a bacterial lawn), bordering (accumulation at the edges of bacterial lawns), and agitated movement in *Caenorhabditis elegans*. This group of behaviors is pronounced in many natural isolates of *C. elegans*, but is relatively weak in other strains, including the standard laboratory strain N2, due to a variant allele of the G-protein coupled receptor encoded by *npr-1* [19]. Several other genes modulating this response have been identified and include genes whose products mediate oxygen sensation, suggesting that social feeding may be a response to hyperoxic environments [20]. In contrast to social feeding in *C. elegans*, the clumping of root-knot nematode juveniles is observed when the nematodes are not feeding. In *C. elegans*, the phenomenon is manifested as loose aggregates with dozens of actively moving L4 or adult animals on an agar plate, whereas the clumps formed by root-knot nematodes contain more individuals and are formed by infective juveniles, an earlier and more uniform developmental stage. A more appropriate comparison may be with the desiccation-induced clumping behavior of certain entomopathogenic nematodes. Infective juveniles of some *Heterorhabiditis* species, but not others, aggregate into large clumps when desiccated at high relative humidity, and this behavior is thought to confer desiccation tolerance [17]. Some plant parasitic nematodes, for example *Aphelenchus avenae* and *Ditylenchus dipsaci*, are full anhydrobiotes, aggregate into large clumps, classically referred to as “eelworm wool,’ and can survive for many years in a dehydrated state [15], [21]. While root-knot nematodes and *Heterorhabditis* species do not have the capability to survive complete drying, clumping may help them to survive moderate levels of desiccation. This, together with our previous observation that clumps disappear when a seedling is introduced nearby [6], suggests that clumping may be a mechanism to increase survival against desiccation or other adverse condition as the J2s await the detection of a suitable host.

Our finding that KCN gradients attract RKNs was initially surprising as cyanide is toxic to many life forms. In fact, cyanide poisoning is responsible for the killing of *C. elegans* by some strains of *Pseudomonas aeriginosa* [11] and has been implicated as responsible for the biocontrol activity of some rhizosphere bacteria against soil pests and pathogens including nematodes [12], [22]. Plant cyanogenesis, that is, release of HCN from damaged tissues, is considered to be a defense against herbivores including nematodes [23], and cyanogenic plant material has also been used for control of nematodes [13]. Potassium cyanide hydrolyzes slowly in a pH-dependent fashion to release HCN, which is highly volatile, resulting in concentration levels that are dynamic and difficult to measure accurately. By our estimates using two different assays, accumulation and clump formation for *M. hapla* VW9, the most responsive strain that we tested, occurs at about 15 µM KCN. We found that movement of VW9 was slowed, but worms were not paralyzed at this concentration. While they were paralyzed by higher concentrations, the paralysis was reversible, even for 110 µM KCN, presumably due to loss of cyanide by volatilization.

A possible explanation for the accumulation of J2 at specific HCN concentrations is that HCN interferes with oxygen perception. *Caenorhabditis elegans* accumulation at particular O_2_ levels is modulated by guanylate cyclases that are expressed in different sensory neurons and balance attraction and repulsion to specific oxygen levels [24], [25]. These soluble guanylate cyclases bind O_2_ or other gasses through associated heme groups, and cyanide, which can bind to heme groups, could compete or otherwise interfere with this signaling. Alternatively, concentration gradients of cyanide may be a natural signal for nematodes as there are many sources of cyanide in the rhizosphere. The formation of clumps at a specific concentration may simply be a consequence of localized accumulation of nematodes as high concentrations of nematodes promote clumping. In fact, other chemicals that cause accumulation, such as weak acids, also result in clump formation when initial concentrations of nematodes suspended in the PF-127 gel are high [7] (Williamson, unpublished). The lower accumulation of *M. incognita* compared to *M. hapla* in response to cyanide may partially explain why *M. incognita* does not form clumps in the presence of CN. This is not due to a generally lower movement rate of *M. incognita* as this species accumulates more rapidly at root tips and at low pH than does *M. hapla* [6], [7]. However it is possible that *M. incognita* is less attracted to cyanide gradients or is more sensitive to paralysis by cyanide.

While clumping behavior appears to be widespread in the phylum Nematoda, closely related species and even strains of a single species differ in their propensity to form clumps [17], [19]. Perhaps each response has an advantage under particular environmental conditions. It may be relevant, for example, that the non-clumping *M. incognita* strain Harmony was isolated from a perennial crop (grape) whereas the other isolates examined were from annual crops. For *C. elegans*, it has been suggested that social feeding and solitary nematodes favor different food dispersal strategies [26]. While variant alleles of *npr-1* explain much of the difference between isolates of *C. elegans*, genetic studies have uncovered several other genes that can modulate the response, and it may be that variant alleles of these genes modulate clumping behavior in other species [25], [27], [28].

The segregation of clumping behavior that we find for our *M. hapla* F2 lines is consistent with the possibility that in this species, as in *C. elegans*, a variant allele of a single gene accounts for much of the variation. However, we do see differences in degree of clumping among the F2 lines suggesting that modifiers of the phenotype may also be segregating in our population. The clumping trait in *M. hapla* is the first phenotype to be genetically mapped in this obligate parasitic species. While the current genetic and physical maps are so far insufficiently developed to identify the responsible genes, positional cloning should become feasible as a greater density map and more F2 lines become available. While transformation is not possible so far for root-knot nematode species, gene silencing by RNAi is an available strategy to assess candidate genes [29]. Identification of genes involved in chemical signaling or responsible for survival in the soil and location and modification of host roots will increase our understanding of this highly evolved parasite and may facilitate the development of novel strategies for control of these damaging pests.

## Materials and Methods

### Nematode cultures

Nematode sources and culture methods are as previously described [7], [30]. Eggs were collected and surface-sterile second stage juveniles were produced as previously described [7].

### Pluronic gel Petri dish attraction assay

Pluronic F-127 (PF-127) (Sigma, St. Louis, MO) gel was prepared as previously described [7]. Plate assays were conducted in standard Petri dishes (100×15 mm). Twenty ml of 23% PF-127 containing the indicated concentration of freshly hatched J2 were poured into each Petri dish at 15°C. Test chemicals were suspended in PF-127 solution and introduced into dispensers made from pipette tips as previously described [7]. Two chemical dispensers were used for each plate. The test plate was observed upside down and pictures were taken under a Nikon SMZ-U dissecting microscope using SimplePCI High Performance Imaging System (Compix Inc, Sewickley, PA). Three or four plates were included in each experiment and each experiment was repeated at least twice. JMP software (SAS Institute Inc., Cary, NC) was used for the statistical analyses. Data were subjected to one way Analysis of variance. Results are reported as significant or non-significant in Tukey's Honestly Significant Difference Test (*P*<0.05).

### Closed tube assay for nematode response to cyanide

Assay set-up is diagramed in Figure 2A. Assays were conducted in tubes produced by cutting clear 5-ml polystyrene aspirating pipettes (VWR, Brisbane, CA) into 52-mm-long sections (assay tubes). Next, 0.6 ml of PF-127 solution (23% w/v in 10 mM sodium phosphate, pH 7.0) was added to assay tubes sealed at one end with parafilm. To produce HCN for the assay, 0.2 ml of 23% PF-127 in 50 mM sodium phosphate, pH 8.0 and containing 0.5 mM KCN was pipetted into a 12 mm tube (donor tube) that was sealed at one end with parafilm. One hour later, the parafilm was removed from the bottom of the assay tube, which was then attached to the top of the donor tube by sealing the junction with parafilm. This joining left an air space between donor and assay gel layers, across which HCN could migrate. A cyanide gradient was allowed to form by incubating at 25°C for 24 h then 0.1 ml of 23% PF-127 solution containing 1300 J2s was added to the distal end of the PF-127 gel. After one hour, 0.5 ml of 23% PF-127 were added to the same end (Figure 2A, image 3) to mark time point 0 of the assay. This experiment was repeated 5 times. Cyanide concentration was measured at several positions within the gel in duplicate tubes without nematodes at time points 1 hr and 24 hr. Five 0.2-ml volumes of PF-127 solution were used for each measurement.

### Cyanide concentration measurement

Cyanide concentration was measured using a colorimetric assay or a cyanide electrode as indicated in the text. A standard pH/mV Meter (Denver Instrument Company, Arvada, CO) was used with a Lazar model ISM-146 Cyanide Combination Ion Electrode (Lazar Research Laboratories) according to the electrode manufacturer's instructions with a few modifications. The cyanide electrode was soaked in 0.5 ml of 0.25 mM KCN PF-127 solution with 0.5-µl indicator solution (0.01% potassium silver cyanide, Sigma) for at least 2 h. A standard curve was generated using 100 µl of 5 µM to 10 mM KCN, prepared in cold PF-127 solution, and 1 µl indicator solution. The standard solutions were kept on ice then transferred to room temperature for 5 min before measuring. After each reading, the electrode was rinsed thoroughly with water for 1 min. Test samples were collected from the indicated region of the PF-127 gel then liquefied on ice. For each measurement, 50 µl solution was used and 0.5 µl indicator solution was added. The standard curve was constructed by plotting mV vs. Log CN concentration. Two samples were recorded and at least 10 readings were made at inner and outer boundary of highest nematode concentration.

The Spectroquant 14800 colorimetric HCN assay (EM Science 14800, Gibbstown, NJ) was used according to the manufacturer's instructions with the following modifications: 1) total reaction volume was reduced to 0.5 ml; 2) solution CN-1A was kept on ice then added to cold cuvettes; 3) cuvettes were kept at room temperature for 5 min before adding CN-2 and CN-3 reagents; 4) readings were taken after 30 min, and 5) reaction mixtures were diluted 1∶1 with dH_2_O before absorbance was read at 585 nm with a DU 640 Spectrophotometer (Beckman Coulter Inc., Fullerton, CA). Experimental samples were measured against the average of 3 controls containing PF-127 gel in10 mM sodium phosphate, pH 7.0. A standard curve was generated using 0.25 to 50.00 µM KCN in PF-127 gel in10 mM sodium phosphate, pH 7.0. Absorbance measurements were fit to a linear correlation model (R^2^ = 0.99).

### Mapping clumping trait in *Meloidogyne hapla* F2 lines

Juveniles of F2 lines previously developed from a cross of *M. hapla* strains VW8 and VW9 [10] were hatched from eggs harvested from greenhouse cultures then surface sterilized. Two or more Petri plates with 300 J2/ml in 23% PF-127 each with two dispensers filled with 2 mM KCN in 23% PF-127 were assessed for each F2 line. At 24 h after assay initiation, the presence or absence of clumps at the opening of the dispenser was scored. The linkage analysis of the clumping trait and polymorphic DNA markers was performed with the software JoinMap 4 [31] using the Kosambi mapping function. Polymorphic DNA markers include AFLP markers [9] and single nucleotide polymorphisms (SNPs). SNP polymorphisms are annotated by the letter ‘C’ followed by the contig number and the nucleotide position of the polymorphism [9] (http://www.pngg.org).
